# Bionanotechnology in Agriculture: A One Health Approach

**DOI:** 10.3390/life13020509

**Published:** 2023-02-12

**Authors:** Mayara Santana dos Santos, Sérgio Antunes Filho, Bianca Pizzorno Backx

**Affiliations:** NUMPEX-BIO, Universidade Federal do Rio de Janeiro, Campus Duque de Caxias Professor Geraldo Cidade, Duque de Caxias 25240-005, Brazil

**Keywords:** agrochemicals, living beings, plant-based diet

## Abstract

Healthy eating habits are one of the requirements for the health of society. In particular, *in natura* foods are increasingly encouraged, since they have a high concentration of nutrients. However, these foods are often grown in the presence of agrochemicals, such as fertilizers and pesticides. To increase crop productivity and achieve high vigor standards in less time, farmers make excessive use of agrochemicals that generate various economic, environmental, and clinical problems. In this way, bionanotechnology appears as an ally in developing technologies to improve planting conditions, ranging from the health of farmers and consumers to the production of new foods and functional foods. All these improvements are based on the better use of land use in synergy with the lowest generation of environmental impacts and the health of living beings, with a view to the study and production of technologies that take into account the concept of One Health in its processes and products. In this review article, we will address how caring for agriculture can directly influence the quality of the most desired foods in contemporary society, and how new alternatives based on nanotechnology can point to efficient and safe solutions for living beings on our planet.

## 1. Introduction

Healthy eating is based on a society’s social and behavioral practices and customs. Therefore, it should encourage the production and consumption of healthy regional foods, such as vegetables, legumes, and fruits. In this context, policies capable of educating and elucidating the direct impact between collective socio-environmental changes and their impact on each individual’s life must be considered. In this way, the link between society, industry, and health policies, strengthening notions associated with quality of life, leads to health promotion and disease prevention [1].

Many aspects must be considered to establish that an individual has a healthy diet. One of the least discussed issues associated with healthy eating habits is linked to food and its cultural meanings. This is due to the fact that an individual needs to adapt their habits to the society they are part of. However, society must also offer subsidies, in order that individuals can establish healthy habits in their routines. In addition, guaranteeing access, flavor, and cost must be a priority for everyone. Fresh foods, such as vegetables and fruits, can be healthy products when cultivated and distributed organically and sustainably. Processed foods, encouraged by marketing, are starting to be replaced by the association of long life and healthy eating [2]. Foods are now seen as protagonists of long life, and healthy and naturally colored foods, as a way to guarantee variety mainly in terms of vitamins and minerals, and considering the cultural and behavioral aspects of a region, are preponderant factors for the new era of food [3].

Another critical point is associated with food safety. Biological or physicochemical contamination and possible health risks must be taken into account. In addition to a healthy diet based on nutritious foods, adequate drinking water consumption and daily physical activities are recommendations associated with a quality life, as the alignment of the circadian clock is called [4].

Environmental awareness has brought new principles to human nutrition, in which the consumption of animals or animal derivatives has been decreasing. A new concept of integrated feeding with living beings then emerges. The plant-based diet is so-called due to the high intake of foods of plant origin. It is based on consuming whole, natural, and minimally processed foods, for example, grains, vegetables, and seeds [5]. A diet based on whole foods and vegetables is a source of good nutrients for the body, such as vitamins, antioxidants, fiber, minerals, and bioactive compounds. In addition, several studies in the modern literature already associate plant-based eating with a lower risk of developing chronic non-communicable diseases, such as type II diabetes, cardiovascular disease, and kidney disease [6,7,8].

Much should be discussed when we associate a plant-based diet and its impacts on environmental sustainability and health, since a plant-based diet has a lower environmental impact than a diet with moderate or high meat consumption. The Lancet EAT Commission in 2019 advocated a population dietary shift toward plant-based food to deal with obesity and climate change, including more vegetables, fruits, nuts, seeds, and whole grains in the daily diet [9].

However, it must be discussed that the increase in plant-based diets can cause stress in the agricultural producers’ market, since this diet is directly associated with seasonality, for example. In addition, the presence of pests in plantations, factors with water stress, and climate change, which are increasingly present on our planet, can lead to a new unbridled trade with the use of agrochemicals (fertilizers and pesticides) that completely diverges from a healthy diet. This is mainly due to the high degree of toxicity [10].

In this sense, bionanotechnology appears as a great ally. It can be an efficient and eco-friendly alternative to agrochemicals that promote regenerative agriculture, integrated with the approach associated with One Health. Bionanotechnology could be applied in agriculture, remediation, water treatment, pest detection, and control, for example, developing porous nano zeolites capable of absorbing water or soil chemical contaminants [11]; nanocapsules capable of releasing the necessary pesticide concentration on demand [12] nanomembranes that control the release of water, fertilizers, and herbicides and serve as water purifiers [13,14]; nanosensors capable of monitoring soil and plants and detecting contaminants and pests [15,16]; magnetic nanoparticles as an agent to combat soil contaminants [17]; among other applications.

Nanotechnology currently enables different alternatives for agriculture, from plant productivity, its interaction with microorganisms, its impact on soil health, and its mechanisms. Through nanotechnology, the scientific world develops new nanomaterials that effectively play the role of agrochemicals, such as nanoparticles (NPs). These NPs can be applied alone or in synergy with plant growth-promoting bioinoculants in different plant organs to replace or minimize the use of agrochemicals [18]. In addition, the NPs have high plant productivity, such as the use of zinc nanoparticles that increase the grain yield of *Pennisetum americanum L.* by 38%, as well as its accumulation of chlorophyll by 24%, the amount of total protein soluble by 39%, and biomass by 12% [19]. This is one of the examples that show the increase in plant productivity caused by NPs. 

The mechanism of action of NPs is being increasingly explored, but there are still many questions about how NPs improve plant growth. It is believed that due to the size of the NPs, which range from 1 to 100 nm, they can more easily enter the pores of the seeds and thus manage to cross the plasma membrane by direct diffusion, endocytosis, or transmembrane proteins. However, this passage through the membrane can be altered depending on the characteristics of the NPs, such as charge, size, morphology, and hydrophobicity, among others. From the entry of NPs into plant tissues, they can be carried by apoplastic or symplastic transport pathways, since NPs can be mobilized from the root part to the shooting part through the xylem and, if distributed through the shooting part, can be translocated through the phloem. Another mechanism of action of AgNPs would be through plasmodesmata and proteins, such as aquaporins. These proteins facilitate the entry of NPs into plant tissues, allowing for the NPs to exert their properties. In addition, aquaporins, ion channels, stomata, and vessels transport NPs throughout the plant [20,21,22].

Understanding the mechanism of action of NPs and reducing the use of pesticides through nanotechnological alternatives can bring many benefits to human beings and the planet, such as increasing food safety, since many foods may contain pesticide residues, reducing the toxicity of fertilizers and pesticides through controlled delivery from NPs, the synergy between bioinoculants that promote plant growth and NPs to replace or minimize fertilizers, among many other examples that will be seen in this article.

## 2. Agrochemicals: Correlation between Environmental Causes and Clinical Problems

Agrochemicals play a vital role in growing food. This importance developed due to large-scale population growth that forced agriculture to increase the production of grains, seeds, roots, leaves, and fruits, in order to feed the population. To achieve this high food production, agriculture has made use of non-renewable natural resources, such as water and land, as well as the excessive use of agrochemicals to maintain crop efficiency [23]. 

Fertilizers are agricultural inputs that increase the productivity and vigor of the crop by promoting the availability of nutrients in the soil for plant growth. Its use is present from large plantations to landscaping [24]. According to data provided by the Food and Agriculture Organization of the United Nations (FAO), in 2000, around 109 kg/ha of inorganic fertilizers, such as phosphorus, nitrogen, and potassium, were used to feed the 6127.7 million inhabitants. In 2014, around 137 kg/ha of inorganic fertilizers were used to feed 7243.8 million people. It is predicted that by 2050 this consumption will increase from 150% to 175%, with a population between 9.4 and 10 million inhabitants [25,26]. Therefore, the forecast is that the use of fertilizers will increase due to population growth. However, there are several problems associated with the excessive use of fertilizers. First, the dose applied per hectare cannot be significantly high. It is necessary to have homeostasis. Otherwise, the beneficial effects will harm the plants, the environment, microorganisms, and living beings.

Pesticides are agricultural inputs capable of killing pests that cause damage or imbalances in plant growth, such as insects, plants, animals, and microorganisms. Its use prevents losses in cultivation and improves the yield of planting and soil use, as its use has become indispensable in agriculture. The term pesticide encompasses various agricultural pesticides, such as fungicides, herbicides, nematicides, and rodenticides, among others [27]. The use of pesticides intensified in World War II (1939–1945) due to food urgency and persists to the present day due to population growth. In the 50s, around 200,000 tons of pesticides were used, and in the 2000s, around 5 million tons were used. As a result, about 3 billion tons of pesticides are used annually in world agriculture [28,29]. Pesticide products occupy about a third of all agricultural input production. To avoid losses and increase crop yields, farmers make excessive use of pesticides, from coating seeds with fungicides to the use of herbicides and insecticides by all plant organs in different periods of the plant cycle. Currently, there are more than 500 different types of pesticides on the market, and many of these products still need to be certified; therefore, they do not have information about the composition and cytotoxicity, since this poses an even more significant clinical and environmental problem [24,26,28,30]. According to the Stockholm Convention, twelve main chemicals are hazardous to health and persistent in the environment. Within this list of twelve products, nine are pesticides [31].

Many soils naturally have the presence of metals, such as Mercury (Hg), Lead (Pb), Arsenic (Ar), Chromium (Cr), Cadmium (Cd), Zinc (Zn), and Copper (Cu), among others. The imbalance between toxic metals and the excess of fertilizers affects, for example, the homeostasis of the soil, causing its acidification in the pH that impacts everything from plant growth to grain consumption. The pH is one of the main parameters of soil health and is crucial for planting. In addition, many agrochemicals have metals and toxic substances in their composition, thus causing physicochemical and biological changes in the soil and plants, such as alteration of metabolic pathways and hormonal signals, degradation of cells and organelles, deterioration in soil use, among other characteristics that can cause loss of planting productivity and poor soil use. From this, there is an excessive entry of metals into the food web, which causes an imbalance in the ecosystem and the health of living beings [24,32,33,34].

It is noteworthy that although zinc and copper are considered heavy and even toxic metals, this may vary according to the mass of these metals that can come into contact with plants or even be ingested by humans. The recommended dietary allowance (RDA) of zinc for adults is 8 mg/day for women and 11 mg/day for men. The tolerable upper intake level (UL) for adults is 40 mg/day, based on a reduction in erythrocyte copper-zinc superoxide dismutase activity. Amounts between 30 and 200 µg Zn g^−1^ dry weight (DW) help plant growth, whereas amounts > 400 µg Zn g^−1^ DW in soil treatments are considered as toxic to the plant [35,36,37]. Regarding Cu, the RDA for adult men and women is 900 μg/day, and the tolerable upper intake level (UL) for adults is 10,000 μg/day (10 mg/day), a value based on protection from liver damage as the critical adverse effect. Furthermore, 5–30 mg kg^−1^ Cu is considered as satisfactory in plant tissues. From the point of view of soil contamination, the Cu threshold value is 100 mg kg^−1^, and the guideline value is 150 mg kg^−1^ [38,39]. In this way, using these metals in agrochemicals or nanotechnologies, such as metallic nanoparticles, can be beneficial or problematic depending on the amount used in soils and plants. In this sense, nanotechnology using a smaller amount of these metals and obtaining results equal to or superior to existing technologies can be one of the advantages of using these more modern technologies. Mainly under the gaze of green nanotechnology.

Environmental issues associated with agrochemicals are intimately integrated with the health of living beings, the environment, and microorganisms. For example, the excess of agrochemicals in the soil is often carried by rain to water reservoirs, rivers, and lakes. Plankton, fish, and all marine life are affected by this contamination from agricultural inputs [27]. Consequently, bioaccumulation and eutrophication, among other environmental causes, will impact other beings in the food web, such as humans and animals that feed on fish [24]. This impacts the world economy with the lower availability of fishing areas, directly affecting the fishing industry, food, and the environment [40]. In addition, humans are affected by consuming contaminated water or food and working directly with these products as farmers. Moreover, some studies report the clinical effects of this contamination by agrochemicals, such as hormonal, reproductive, immunological, neurological, respiratory, carcinogenic problems, and even behavioral disorders [41,42,43,44]. The entire human physiology is compromised from exposure to agrochemicals. Furthermore, there has been a generalized loss of homeostasis for generations, whether by people who are physically ill, such as cancer, or mentally ill, due to depression and psychological disorders [42]. For example, women who have had direct or indirect exposure to agrochemicals are more likely to develop breast cancer [45], and pregnant women have about a 30% chance of having children who develop childhood brain cancer [46] and a greater chance of having children with leukemia [47]. In addition, the fetus may present cardiac and neuronal problems and morphological alterations in the lower and upper limbs [40]. Men may have a 40% risk of developing prostate cancer compared to men who have not had contact with agrochemicals [48]. It is noteworthy that exposure to agrochemicals at any stage of life, from the womb to old age, can lead to chronic problems that develop in the long term, such as Parkinson’s and Alzheimer’s disease [49,50].

Another issue is that if planting is started in contaminated soil or with a seed coated with a toxic fungicide, the plant will start germinating and absorb the water available in the soil along with metals and toxic substances. By absorbing these toxic inputs, they can be internalized in plant cells causing susceptibility to abiotic stresses or even enter the meristem, which is responsible for the cell differentiation of plant tissues. Suppose the toxic input is internalized to the meristem, in this case, all differentiated plant cells can contain their presence, and it can be carried throughout the plant cycle from seed germination to the consumption of grains, fruits, flowers, and roots, among other vegetable organs. Moreover, this can be even worse with the addition of pesticides during plant development. In this way, the animal that consumes this food can be contaminated with these toxic inputs, altering its microbiota and metabolic regulations. 

Farmers’ exposure to the application of agrochemicals is dangerous for their health in several aspects. Cardiovascular diseases are directly related to this exposure, which can lead to myocardial infarction, congestive heart failure (CHF), stroke, arrhythmia, and sudden death. The oxidative stress of these agricultural inputs, combined with their transport through the blood, makes the heart one of the organs most exposed to the risks of agrochemicals [51]. Different places in the world, such as Thailand and Zimbabwe, have studies that show the prevalence of organophosphate poisoning on the crescent exposure of the farmer to agrochemicals, in addition to bioaccumulation of pesticides on the clothes of those who apply the product, inhalation, and direct contact with the skin when there is no correct use of individual protection equipment [52,53]. Furthermore, adjuvants used in agrochemical application sprays may not be safe, as in the case with organosilicons surfactants. Other types of surfactants may be vehicles for greater dissemination of toxic compounds to the environment and the farmer, as well as raising the level of toxicity by carrying and mediating the contact of organisms with the compounds [54].

**Figure 1:**
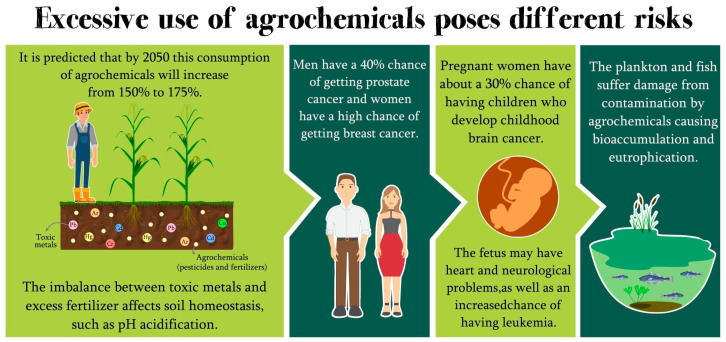
Excessive agrochemicals can present environmental, human, and animal risks.

Therefore, the human being that consumes this animal will also present the toxic effects of agrochemicals (Figure 1) [55,56,57]. Following this rationale, we will see how agrochemicals can impact living beings through the One Health vision in the following topic. 

## 3. One Health Approach

In recent decades, several studies have sought to understand the impact that technologies used in different social areas and production chains can have on the production environment, the product itself, and, finally, the final consumer, especially when this product is related to food consumption [58]. The term “One Health” was used for the first time in 2003–2004 at a conference at Rockefeller University, where the world’s health was the main topic, given that the impact that technology has on a living being of a given species can impact other genera, families, and kingdoms [59,60]. This approach is related to agrochemicals when we observe several studies that indicate an impact of these inputs on the health of animals, humans, and the ecosystem, since, in addition to the food web, all living organisms are affected in agriculture as well as ecosystems of plantations [61]. More recently, the Food and Agriculture Organization of the United Nations (FAO), the World Organization for Animal Health (OIE), the United Nations Environment Program (UNEP), and the World Health Organization (WHO) are forming operational guidelines. For a better understanding and definition of the term “One Health” and how the countries and industries of the world can contribute to more sustainable approaches to improve the quality of life of human beings, following the tripartite is elaborated according to One Health: The communication between the practical actions related to the concept, society itself, and the sectors, in order that there are collaborations and training about this approach around the world [62]. 

**Figure 2:**
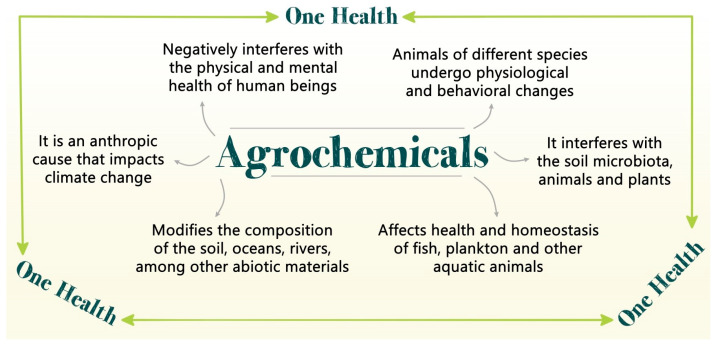
Agrochemicals and their impacts from the point of view of the concept of One Health.

The agrochemical inputs used in plantations negatively or positively impact microorganisms and aquatic and terrestrial animals, and the impacts are desired exclusively for agricultural production. Within this scope, one of the significant challenges is biomonitoring to understand the full impact that agricultural inputs, mainly agrochemicals, can have on all living beings and the environment. With the constant advancement of biomonitoring technologies, it will be possible to understand even better the impact of pesticides and inputs of different chemical natures, such as the toxicity of metals, such as Cd, Pb, Cu, and Zn, and how the performance of agrochemicals influences the ecosystem from the beginning. Plants, soil, and human and animal health are consumers of food produced [63,64]. Similarly, several other inputs with varied chemical natures impact the water table, the farmer, and the final consumer. This use of substances is harmful and toxic for human physiology, but also for the microbiota of the soil, in which the planting carries out the microbiome of the plants and the microbiota of the final consumer. The chemical compounds used in agriculture can select the microorganisms (bacteria and fungi) that will remain in the soil. This can impact the growth of plants planted on this land to the final consumers of agricultural production, such as humans and animals. Soil and plant microbiota can be affected by herbicides, fungicides, and insecticides. For example, herbicides, such as glyphosate, suppress the activity of phosphatases and can reduce the growth and activity of *azotobacter*; fungicides, such as Captan and Thiram, decrease the growth and nitrogenase activity of *Azospirillum brasilense*; insecticides, such as Methamidophos, reduce the microbial biomass by 41–83% [65]. Another issue relevant to pathogenic microorganisms in plants, humans, and animals is the ability of agrochemicals to carry out constant selective pressure and maintenance of resistant strains, even stimulating the horizontal transfer of resistance genes and the interspecific dissemination of antibiotic resistance genes [66]. Environmental exposure and pesticide concentrations used in plantations can stimulate and diversify the evolution of *E. coli* until reaching high levels of resistance to different classes of antibiotics. In addition, mutations caused by agrochemicals affect the regulation of gene transcription related to the formation of bacterial biofilms and defense against oxidative stress, among other mechanisms that make bacterial cells more resistant to antibiotics [67]. In addition to microorganisms, terrestrial animals are directly affected, as in the case with bees, which are vital pollinators for the ecosystem. These animals can suffer multiple stresses, including exposure to low levels of agrochemicals, which increase their mortality rate [68]. Moreover, the honey produced by bees that feed on the nectar of flowers in areas exposed to agrochemicals is altered, and the animals that consume this honey may suffer from its toxicity, which is directly related to the impact on the reproduction of their species [69]. Aquatic animals, such as fish, are also affected by both toxic metals and compounds directly and indirectly, which can affect the development of these animals, mainly in more sensitive species, and the early stages of life and, in addition, the bioaccumulation of heavy metals affects the entire the food web [70,71]. In this way, it can be observed that the entire ecosystem can be impacted by chemical treatments carried out in agriculture, and for the farmer, it is no different. In addition, the human gut microbiota is severely affected by pesticides directly [58], and gastrointestinal disorders and neural diseases, including neurodegenerative diseases, such as Parkinson’s or Alzheimer’s, are more likely to develop with the consumption of foods containing agrochemicals [72,73]. This can also be of concern for producing new foods and for vegan, raw food, and vegetarian people who consume more plants and vegetables [74]. Therefore, many technologies used in agriculture cause a network of consequences and problems that go directly against the concept of One Health (Figure 2).

## 4. Bionanotechnology and Agriculture

Bionanotechnology is a synergistic joining of two multidisciplinary sciences, nanotechnology and biotechnology. Nanotechnology is an interdisciplinary and transdisciplinary science that studies the manipulation and improvement of matter at atomic and molecular scales. The prefix “nano” has an etymological origin in the Greek language which means dwarf. This prefix justifies the dimension of the nanometer, which corresponds to 1 billionth of a meter, thus, 10^−9^ m. The sizes of nanomaterials, such as NPs, follow a nanometric scale ranging from 1 to 100 nanometers [75]. Due to the nanoscale, it is possible to develop new technologies, services, and products with differentiated structures and properties that other scales do not have.

Furthermore, biotechnology is a multidisciplinary science that relates different areas of knowledge, such as biochemistry, genetics, microbiology, physiology, immunology, food engineering, and agronomy, among others. Therefore, biotechnology relates a set of knowledge, techniques, and scientific methods to solve environmental, biological, and medicinal problems through technological and innovative bias [76]. From these concepts and application possibilities, bionanotechnology can be applied as an ally in agriculture to replace and minimize agrochemicals, biomonitoring, and develop agricultural nanosensors.

Due to the importance of biomonitoring for understanding the impact of agrochemicals on the ecosystem and keeping in mind the concept of One Health, nanosensors can be crucial to understanding the fate of these chemical compounds used in agriculture and how these residues are deposited along the way in the food web—enabling the analysis of interspecific bioaccumulation in a more accurate way, including the bionanotechnologies used in plants [77,78]. Understanding and monitoring soil conditions and plant development, such as nutritional and hormonal pathways, is essential for the treatment with nanomaterials. From this, it is also necessary to understand the nanomaterials’ cytotoxicity, from the plant cells to the organisms of different animals, such as fish, bees, and humans, among others. With this, it is possible to predict and avoid the bioaccumulation of nanomaterials and the generation of another toxic technology for the ecosystem. Another issue is that investigating the delivery of nanomaterials through nanosensors by all plant organs helps in the construction of technologies with targeted mechanisms of action, in the biosafety of use and consumption, and makes it possible to understand the life cycle of the nanomaterial from its planning to the final destination [79,80]. Nanotechnology can contribute to sustainable agriculture in several ways, including the use of NPs, quantum dots, nanorods, nanoencapsulation, and nanoemulsions, among other products that reduce the concentration of chemical compounds, which can be harmful and are already used on a large scale, or avoid its use both in the synthesis process and in the product used, in order to reduce the impacts on living beings and the ecosystem [78]. 

The use of nanomaterials, such as metallic nanoparticles and metal oxide nanoparticles, has gained prominence in agriculture in recent years due to their multidisciplinary properties and applications ranging from seed production to food packaging [81]. Among the different possibilities of synthesizing nanomaterials through biosynthesis or green synthesis, it has been the most efficient method due to its approach that minimizes or eliminates the use of compounds, processes, and concentrations of reagents, which may be toxic to human health and for the environment [82]. Therefore, the green synthesis of NPs, nanoemulsions, and nanoencapsulation has been used to reduce the use of fertilizers, increase crop productivity, improve crop management, and protect seeds [82,83]. As a result, several bionanotechnological techniques aim at plant improvement without genetic modification or with the lowest use of bioinoculants, and thus present low cost, dynamic synthesis, and agrochemical reduction.

Nanoscale drug delivery can be engineered with different materials and for varied purposes, from targeting important biomolecules for metabolic processes and enhancing plant growth to therapeutic effects and disease treatment. Mesoporous silicon-based nano-carriers (MPSNPs) can carry macromolecules, such as proteins, enzymes, or even antibodies on their surfaces. Solid lipid nanoparticles (SLN) can carry antioxidant molecules, natural antimicrobial compounds, and hydrophobic agents that favor plant growth. Nano-capsules, micelles, liposomes, nanoemultions, dendrimers, nanocrystals, nanogels, and other nanotechnologies are being studied and developed as drug delivery systems in agriculture [84]. 

Another exciting technology is using polymeric nanocarriers made from natural or synthetic polymers that are biocompatible and biodegradable. The stability of these carriers in the wood sap takes place through the colloidal stability of these nanosystems, the biodistribution being addressed by binding biomolecules in tissue-specific receptors or general receptors, in order that the bioactive molecule reaches all tissues efficiently [85]. In this sense, the supply of nutrients for the biofortification of plant cultures can be carried out with carriers made with cellulose nanofibers, such as nanofibers, polymer-nano cellulose-clay composites, nanocarriers derived from silk fiber, and carboxymethylcellulose. These carriers can supply the deficiency of plant nutrients or improve their growth and development [86].

Some nutrients are impressive for the plant life cycle, such as phosphorus, although they are healthy, among others. Phosphorus, for example, is a vital element for all plants, as it is essential for growth, photosynthesis, protein structure, and the formation of nodules for flow formation, among others. [87]. In cereals and legumes, the primary storage form of phosphorus is in the form of phytic acid. In this form, plants absorb phosphorus less due to the fact that the phytic acid can form insoluble complexes with proteins and minerals [88]. This is an example of several others that occur in agriculture. To solve this problem found in phosphate fertilizers, nanotechnology has been used as nano fertilizers to modulate phosphate solubility through hydroxyapatite nanoparticles that release phosphorus in a controlled manner, reducing bioaccumulation and formation of insoluble complexes [89,90]. In a study with nanoparticles of hydroxyapatite coated with urea, they showed an increase in the germination rate, in the aerial part, and in the dry mass of chickpea seedlings (*Cicer arietinum*) [91]. Similarly, there are several studies that investigate the possibility of nanofertilizer in maize (*Zea mays*) [90], soybean (*Glycine max*) [92], lettuce (*Lactuca sativa L.*) [93], among other cultures. In this way, nanotechnology proves to be a promising technology for solving problems that occur in translational fertilizers.

Among the different areas of nanotechnology in agriculture, seed treatment is increasingly investigated by science. By treating the seeds, the vegetable is expected to start its cycle with greater productivity, vigor, and less agrochemical toxicity during and after cultivation. For example, the seed nano-priming technique using NPs and the seed bio nano-priming technique using plant growth promoting bacteria (PGPB) in synergy with NPs are promising technologies for agriculture [94]. This seed priming methodology accelerates the imbibition phase and the induction phase of germination growth without the pre-initiation of the phase of embryonic axis growth [79]. This allows the farmer to initiate germination at different times. From the development of the embryonic axis growth, it is expected that the seedling will obtain improved growth and may have more resistance to different abiotic and biotic stresses [81]. With this, there is a reduction in the use of agrochemicals, from the coating of fungicides on the seeds to the use of insecticides and herbicides throughout the plant’s life cycle, due to the controlled delivery of nanomaterials [79]. Furthermore, by joining nanomaterials with PGPB, a fertilizing effect is expected for the plant, as well as more excellent resistance to diseases caused by biotic and abiotic stresses [95].

For example, one can cite chitosan nanoparticles (nanochitosan) in synergy with PGPB (*Pseudomonas taiwanensis* and *Pantoea agglomerans*) in maize seeds. This synergy between nanomaterials and a consortium of bioinoculants resulted in an increase in photosynthetic pigments by 65.62%, in the number of leaves by 67.18%, in plant height by 54%, and in the flavonoid content by 167.61%. In addition, there was an increase in the activity of antioxidant enzymes, such as catalase at 80.15% and peroxidase at 25.25%, which is an essential characteristic of stress tolerance [96]. In another study, the growth and soil health of maize seeds was treated with CSNPs in synergy with PGPB Bacillus spp. (bacterial isolates PS2 and PS10). The work showed that treatment with nanochitosan increased plant height and leaf area. These characteristics can be attributed to the action of plant hormones. The nanochitosan also induced seed germination. This may be related to the increase in seed permeability that allows for a greater entry of water and oxygen into the cells, thus accelerating metabolism. In the same study, the authors evaluated soil samples after treatment and observed that the amount of available phosphorus increased. There was also an increase in alkaline phosphatase enzyme activity after the treatment with nanochitosan, indicating a greater amount of substrate available in the soil. Therefore, the increase in enzymatic activity in the soil can be an indicator of soil health, since this can be related to an increase in microbial biomass [97,98]. Another study investigated the impact of a liquid formulation of nanophosphorus and phosphate-solubilizing bacteria called nanophos on maize crops. The soil treated with nanophos increased the shoot and root part and the number of leaves of the plant. In addition, there was an increase in the protein content in the leaves and the activity of antioxidant enzymes. This formulation increased the activity of enzymes in the soil, such as phosphatase, arylesterase, and β-glucosidase, among others [99].

Another example is using iron oxide nanoparticles (FeONPs) biosynthesized from *Syzigium cumini* leaf extract in synergy with *Rhizobium pusense* in *Vigna radiata* plants. The bionanofertilizing potential of this interaction showed a significant increase in the length of the shoot (49%) and root (7%) and dry biomass of the shoot (21%) and root (80%). Furthermore, proteins present in *Vigna radiata* seeds after the treatment with *R. pusense* and FeONPs increased by 42% compared to the untreated control plants. The amounts of proline and chlorophyll were also increased by around 37%. It is noteworthy that this interaction did not cause damage to the membranes and tips of the roots [18]. In another study, bionanofertilizers were synthesized from *Pseudomonas gessardi* and *Pseudomonas azotoformans* and nanocomposites formulated from soy and cerium oxide (Ce) for application in *Trigonella foenum-graecum*. This study showed that NPs significantly increased shoot and root height without Ce bioaccumulation in the soil [100]. In another study, the synergy between silica nanoparticles (SiO_2_NPs) and the PGPB *Bacillus cereus* in *Capsicum annuum L* was shown. Studies show that SiO_2_NPs increased phosphate solubilization and production of the plant hormone gibberellin due to the potentiation of *Bacillus cereus* growth. This interaction also increased the antioxidant enzymes catalase and superoxide dismutase, indicating that SiO_2_NPs induce defense-related responses. In addition, there was an increase in shoot and root length, number of leaves, and number and production of fruits [101]. More examples can be seen in Table 1.

In addition to the interaction of NPs with PGPB, recent studies explore nanomaterials’ influence on plants. For example, the use of zinc oxide nanoparticles (ZnONPs) as biostimulants to mitigate drought and high salinity in rice and wheat crops, respectively [110,111]. The use of silver nanoparticles (AgNPs), titanium dioxide nanoparticles (TiO_2_NPs), magnesium oxide nanoparticles (MgONPs), and magnetic nanoparticles to minimize the use of fungicides and bactericides in seeds, leaves, and roots, as well as herbicide/insecticide actions in tomato, maize, wheat, among other crops of significant economic and food [112,113,114,115,116]. More examples of the effects of nanomaterials on plants can be seen in Table 2. Moreover, bionanotechnology is already present in many formulations available on the agricultural market, such as the organic nanoemulsion marketed by the company Vision Mark Biotech called Nano Guard. This nanoemulsion is a pesticide based on natural extracts with particles smaller than 200 nm that present stability and prolonged action due to its controlled release. Moreover, Tropical Agrosystem nanoemulsion formulates its pesticides from natural extracts and moves the pesticide market with revenues of more than USD 50 million annually. These examples confirm the importance of bionanotechnology in developing new agricultural alternatives that can reduce the use of agrochemicals with compositions that are harmful to health and the environment [117]. 

## 5. Conclusions

The consequences due to the excessive use of agrochemicals impact all beings in the ecosystem, from changes in the microbiota to morphophysiology and body homeostasis. All living beings are directly or indirectly affected, causing severe economic, social, and environmental damage, especially considering the emerging causes of climate change that accentuate various problems for our planet. Therefore, many issues need to be investigated using the One Health concept and agriculture. Bionanotechnology can solve these problems through nanosensors, nanoparticles, and agricultural nanoformulations. It is also worth mentioning that studies involving bionanotechnologies and agriculture are recent and need further explanations, ranging from the mechanism of action in different stages of plant development to the final destination of metabolization in the soil and living beings. Therefore, bionanotechnology is expected to positively impact living beings, mitigate problems related to climate change, improve crop management and, above all, reduce the use of agrochemicals and the risk of global contamination. The hope is that bionanotechnology can be an agent of regeneration in all stages of life.

## Figures and Tables

**Table 1:** Examples of the interaction between plant growth-promoting bacteria and nanomaterials in plant organisms.

Nanomaterial	Crops	PGPB	Effect	References
Nanozeolite	*Zea mays*	*Bacillus* spp.	Increase in the chlorophyll and carotenoid content, in the total sugar content, in the protein content, in seed germination, in the total phenolic content, in shoot and root height. The number of leaves and leaf area also increased significantly.	[102]
Nanotitania	*Triticum aestivum* L.	*Alcaligenes faecalis* *Paenibacillus polymyxa* *Bacillus thuringiensi*	Biomass increase under conditions of saline stress and pathogenic stress by the fungus *Fusarium culmorum*.	[103]
Nanosilicon dioxide	*Zea mays*	*Pseudomonas taiwanensis* *Pantoea agglomeranscom*	Increase in the plant’s height, the number of leaves, the content of chlorophyll, soluble proteins, phenols, flavonoids, and carotenoids. In addition, there was an increase in the antioxidant enzymes, catalase and peroxidase, and an improvement in soil health.	[104]
ZnONPs	*Cucumis melo*	*Bacillus fortis*	The synergy between PGPB and ZnONPs improved cadmium phytotoxicity. In addition, there was an increase in biochemical and plant growth parameters.	[105]
ZnONPs	*Solanum lycopersicum* L.	*Bacillus subtilis* *Lactobacillus casei* *Bacillus pumilus*	Increase leaf width, plant height, and fresh and dry weight of leaves and roots under saline stress conditions. Additionally, the synergy between PGPBs and NPs resulted in decreased DNA methylation.	[106]
FeONPs	*Trachyspermum ammi* L.	*Providencia vermicola*	The synergy between PGPB and FeONPs resulted in increased plant growth, photosynthetic pigments, sugars, and biomass under heavy metal arsenic stress conditions.	[107]
CuONPs	*Triticum aestivum* L.	*Bacillus subtilis* *Lactobacillus casei* *Bacillus pumilus*	Treatment with CuONPs and PGPB increased wheat tolerance to saline stress and showed an antigenotoxic effect.	[108]
Nanochitosan	*Zea mays*	*Pseudomonas taiwanensis* *Pantoea agglomeranscom*	An increase in seed germination, vigor, average height, carotenoid, and chlorophyll content. In addition, nanochitosan can improve beneficial bacterial growth.	[109]

**Table 2:** Examples of the effects of bionanotechnology on plant health and agricultural productivity.

Nanomaterial	Crops	Effect	References
MgONPs	*Gossypium hirsutum*	Increase in plant height, due to Mg modulating the transport of sap elaborated by the phloem to younger leaves, and in the size and number of leaves, due to activation of the enzyme RuBP carboxylase and assimilation of proteins and carbohydrates.	[118]
MgONPs	*Zea mays*	Increase in shoot and root length, germination rate, and plant vigor due to the biostimulant effect of MgONPs. Increased chlorophyll and carotenoid content at lower concentrations of MgONPs.	[119]
FeONPs	*Oryza sativa*	FeONPs increased germination speed, water absorption, and stimulation of α-amylase enzyme and total sugars. In addition, they showed antioxidant effects from tests carried out with the enzymes peroxidase, catalase, and superoxide dismutase.	[120]
FeONPs	*Triticum aestivum*	FeONPs mitigated salt and Cd stress in wheat. The NPs made available in the soil increased the presence of nutrients, such as N, P, and K^+^ and reduced the concentrations of Na^+^ and Cl^-^ in wheat grains and Cd in the soil.	[121]
FeONPs and ZnONPs	*Triticum aestivum*	The NPs helped wheat in the abiotic stress caused by Cd. NPs decreased the activity of superoxide dismutase and peroxidase enzymes in leaves and reduced the concentration of Cd in the root and aerial parts of wheat.	[122]
ZnONPs	*Oryza sativa*	ZnONPs increased the number, width, and height of the leaves, the length of the root and aerial part, the vigor, and the fresh and dry mass of the seedlings. This increase is due to the absorption and translocation of the zinc content by the rice grain.	[123]
ZnONPs	*Solanum melongena*	ZnONPs increased productivity and fruit growth under water and salt stress conditions by 22.6% at higher concentrations (100 ppm) and 12.2% at lower concentrations (50 ppm).	[124]
TiO_2_NPs	*Phaseolus vulgaris*	The penetration of TiO2NPs allowed for the uptake of water inside the seeds, significantly increasing the length of the aerial part and root part. Increased activity of antioxidant enzymes catalase and peroxidase.	[125]
TiO_2_NPs	*Salvia officinalis*	TiO2NPs increased dry mass by 30%, water use efficiency by 35%, and net profit rate by 44% in salvia plants under water stress.	[126]
CuNPs	*Solanum lycopersicum*	CuNPs increased the content of vitamin C (80%), phenols (7.8%), glutathione (81%), and antioxidant enzymes, such as superoxide dismutase and ascorbate peroxidase in tomatoes under saline stress conditions.	[127]
Nanochitosan	*Catharanthus roseus*	Decrease in the accumulation of malondialdehyde and H_2_O_2_, and preservation of membrane integrity in conditions of water stress. CSNPs induced alkaloid biosynthesis gene expression and increased total chlorophyll concentration and stomatal conductance.	[128]
AgNPs	*Triticum aestivum*	AgNPs increased relative water contents by 12.2%, membrane stability index by 26.5%, chlorophyll by 10%, chlorophyll b by 16.4%, and total chlorophyll by 19% in wheat plants under heat stress.	[129]
Nanogypsum	*Spinacia oleracea*	Nanogypsum can mitigate the effects of salinity-sodicity and increase spinach productivity in saline-sodic soil. The nanomaterial reduced soil salinity by 83%, fresh mass, leaf area, and plant height.	[130]

## Data Availability

Not applicable.
